# vNOTEsHC : Hysterectomy by transvaginal natural orifice transluminal endoscopic surgery versus laparoscopic for large uteri: study protocol for a multicentre randomised controlled trial

**DOI:** 10.52054/FVVO.15.3.083

**Published:** 2023-09-24

**Authors:** J Druenne, E Presles, T Corsini, S Campagne Loiseau, S Curinier, A Mansour, G Lamblin, Q Reboul, C Chauleur

**Affiliations:** Univ Jean Monnet, Department of gynaecologic and Obstetrics, University Hospital of Saint-Etienne, France; Univ Jean Monnet, URCIP; Department of Gynaecology and Obstetrics, University Hospital of Clermont-Ferrand, France; Department of Gynaecology and Obstetrics, University Hospital of Issoire, France; Department of Gynaecology and Obstetrics, University Hospital of Lyon, France; Department of Gynaecology, Clinique Mutualiste, Saint-Etienne, France; INSERM, U 1059, 42023, Saint-Étienne, France

**Keywords:** Hysterectomy, benign indications, large uterus, vNOTES, laparoscopy

## Abstract

**Background:**

In France, 62,000 hysterectomies are performed per year, 70% of which are benign. The choice of approach (laparotomy, laparoscopy or vaginal route) is particularly important in the case of large uterus (> 280g) which are associated with a higher risk of complications. The current data are not sufficient to favour one or other approach. A new medical device, the vNOTES (Natural Vaginal Orifice Transluminal Endoscopy System), offers the advantage of both laparoscopic and vaginal route for pelvic surgery.

**Objectives:**

To demonstrate the superiority in terms of intraoperative and postoperative complications of the use of a natural orifice transluminal endoscopic hysterectomy system (vNOTES) versus laparoscopic hysterectomy for benign pathologies on estimated large volume uteri (>280g)

**Materials and Methods:**

A randomised, double-blind, superiority trial will be performed at five hospital centres. Women with benign uterine pathology requiring hysterectomy and with a large uterus (> 280g) will be randomised to receive either laparoscopic or vNOTES hysterectomy.

**Main Outcome Measures:**

The primary outcome will be the occurrence of intraoperative and postoperative complications within 6 weeks of surgery. Secondary outcomes will be conversion during surgery, duration of surgery and hospitalisation, postoperative pain, postoperative complications, resumption of sexual life and satisfaction with the surgical team.

**Results:**

248 women will be randomised.

**Conclusion:**

This trial will provide a better understanding of the approach to large uteri optimise the care of these thousands of women undergoing hysterectomy.

**What's new?:**

This trial will evaluate the vNotes for large uteri.

## Introduction

In France, there are 62,000 hysterectomies performed per year, of which 70% are for benign indications (Lee et al., 2019).

The choice of surgical approach (laparotomy, laparoscopy or vaginal route) is particularly important in cases with large uteri (> 280g) as these are recognised to be associated with a high risk of complications and conversion. Currently minimally invasive approaches are recommended by the Collège national des gynécologues et obstétriciens français (CNGOF) (grade C) (Deffieux et al., 2015).

The vaginal and the laparoscopic route have their advantages and disadvantages.The latter is associated with an increased rate of conversion to laparotomy and post operative complications compared with vaginal hysterectomy (15% for the vaginal route, 37.5% for laparoscopy) and is particularly true for large uteri > 280g (Lee et al., 2019; Daraï et al., 2001; Gendy et al., 2011).

A Medical Device, the vNOTES (Natural Vaginal Orifice Transluminal Endoscopy System), offers the advantage of minimally invasive surgery by endoscopy through the vagina. Several feasibility studies in gynaecological surgeries have already been performed (Baekelandt, 2018a; Baekelandt, 2018b; Baekelandt et al., 2018c; Lowenstein et al., 2020; Baekelandt, 2019). Randomised trials have shown that the vNOTES allows hysterectomies to be performed without conversion with less pain, fewer postoperative complications and a shorter hospital stay when compared to laparoscopy (Baekelandt et al., 2019; Baekelandt et al., 2021). More recently, the developers of vNOTES found favorable results for vNOTES in terms of conversion and complication rates (Nulens et al., 2021) in large volume uteri (>280g).

The main objective of our study is to demonstrate the superiority in terms of intra and post operative complications within 6 weeks after surgery with the use of a vNOTES hysterectomy versus laparoscopic hysterectomy for benign pathologies on estimated large volume uteri (>280g).

## Material and Methods

### Type of study

This is a prospective, open-label, multicentre, randomised, parallel-group superiority study stratified on centre and uterine volume (280-500g; >500g). It evaluates conversion and intraoperative and postoperative complications within 6 weeks of surgery using the transluminal natural orifice vaginal endoscopic system for hysterectomies (vNOTES) versus laparoscopic hysterectomy.

### Population of study

The study will take place at the University Hospital of Saint-Etienne in the gynaecology- obstetrics department, at the Clermont Ferrand University Hospital and at the Issoire University Hospital, at the HCL HFME in Lyon and at the Clinique Mutualiste in Saint-Etienne.

This study is intended for any woman who is seen in a preoperative consultation in the Gynaecology-Obstetrics department of an investigating centere for a benign pathology of a large uterus requiring a hysterectomy.

The inclusion criteria will be: patients over 18 years old, affiliated or entitled to a social security system, having given their agreement to participate and after signing the consent form, presenting with a benign pathology of the uterus requiring a hysterectomy and having a large uterus estimated at more than 280g. A uterus will be defined as large if it corresponds to a gravid uterus of 12 weeks size, the 2D ultrasound or MRI measurements estimate the volume >280g with the formula LxWxAP, or by measurement of a volume in 3D ultrasound with the software VOCAL.

The exclusion criteria will be non-French- speaking patients, those who have not given their consent to participate in the study,are subject to a legal protection measure, unable to express their consent or those with a contraindication to the use of vNotes (endometriosis).

This study is currently being reviewed by the ethics committee.

### Sample size calculation

Based on the figures found in two studies on large uteri (Daraï et al., 2001; Hwang et al., 2002) and Baekelandt’s study of hysterectomies (Baekelandt et al., 2019), for an alpha risk of 5% and a power of 90%, a superiority study with an expected complication rate of 37% with laparoscopy and 15% with the vaginal route (i.e. an RR of 0.5 and therefore a relative reduction in risk of 50%) will need to include 119 patients per group, i.e. 236 in total. In order to compensate for possible loss of sight, 248 patients will be included.

### Study design

An information brochure on the study will be given to all patients who meet the inclusion criteria of the study at the preoperative consultation. An evaluation of pain by EVA scale will be carried out during this consultation.

After obtaining the patient’s consent on the day of the operation, the allocation between the two groups will be done by randomisation. This randomisation will be stratified on the centre and uterine volume (280-500g; > 500g). The randomisation list will be a randomised computer list established before the beginning of the study. It will be balanced by blocks of variable size and centralised via the Ennov Clinical web platform.

Two groups of patients will be constituted: a “laparoscopic approach” group and a “vNOTES” group. Patients will not know their group of assignment.

All hysterectomies will be performed by vNOTES-trained surgeons (20 procedures performed) who will follow the standard operating procedure. In women assigned to the experimental arm, the surgeon will perform a hysterectomy with the vNOTES medical device. In the women assigned to the control arm, the surgeon will perform a laparoscopic hysterectomy.

A questionnaire assessing visual comfort, feeling of safety and satisfaction on Lickert scales will be completed immediately after surgery by the operator and the operating assistant.

The care provided by the anesthetists and nurses will be standardised and similar in both groups. To limit bias, in all cases the patient will have abdominal dressings as if it underwent a laparoscopy.

Once returned to the ward, all participants will be assessed every 2 hours and discharged home when the patient’s condition allows(Chung score ≥ 9/10).

Telephone interviews by the department CRA, blinded to the allocation group will be conducted daily for the first week to assess postoperative pain.

A follow-up visit by an independent adjudication committee, blinded to the allocated group will be performed 6 weeks postoperatively or earlier if a complication brings a patient to consult.

Finally, a telephone call will be made at 3 months to evaluate the sexual impact by a questionnaire (PISQ12 with a score ranging from 0 to 48) (Table I).

**Table I:** Conduct of the study.

Visits	V1	V2	V3	V4	V5	V6
Title of the visit	Pre-operative selection visit	Day of the intervention	Immediate post-operative follow-up	Telephone follow-up D1-D7	Post-operative visit at 6 weeks	Questionnaire at 3 months
Information	X					
Eligibility Criteria	X					
History of comorbidities	X					
Consent		X				
Randomisation		X				
Intervention*		X				
Adverse events		X	X	X	X	
Chung's score **			X			
Post-operative pain assessment ***			X	X		
Clinical examination ****					X	
Sexuality Questionnaire						X

X specific acts to research; *According to the allocated group; **Chung score; ***by scale from 0 (no pain) to 10 (maximum pain); ****standardised clinical examination: general condition, BP, abdominal palpation, vaginal examination.

The study protocol has been validated (N° clinical trial : NCT05884658).

### Outcomes

The primary outcome is a composite endpoint including the occurrence of intraoperative and postoperative complications within 6 weeks of surgery. Intraoperative complications will be defined as injury to surrounding organs (bladder, ureters, rectum, colon, small intestine) or bleeding requiring immediate resumption, intraoperative transfusion and/or decreasing the patient’s haemoglobin by more than 2 points compared to the last known pre-operative blood test. Postoperative complications will be defined according to the Clavien-Dindo classification.

Regarding secondary outcomes, the need for conversion during surgery will be collected.

Post-operative pain at day 0 will be evaluated by the maximum daily pain felt on arrival in ambulatory surgery and at hour 3 after return from the block. Short-term postoperative pain during the first week after surgery will be measured by a numerical scale (0 to 10), determined on a daily telephone call. The consumption of analgesics other than those planned in the study in the immediate postoperative period and within 7 days will also be recorded. The duration of postoperative reflex ileus will be measured between the day of the operation and the resumption of a transit (appearance of the first gas) in hours. The duration of the operation from the incision to the end of the closure (in minutes). The duration of hospitalisation will be evaluated in days and the possibility of returning home by the time in hours between the surgical incision and the achievement of a Chung≥ score of 9/10. Sexual disorders: sexual life will be assessed by the difference in the specific validated score (self-questionnaire) (PISQ12 with a score ranging from 0 to 48) between the inclusion date and 3 months after surgery for sexually active women. The visual comfort, satisfaction and feeling of safety of the surgeon and the operating assistant will be evaluated via a Likert scale.

### Data collection

Demographic data including age, weight (kilograms), height (cm), BMI (Kg/m2), medical, surgical and obstetric history will be collected directly in the observation notebooks as the study visits take place.

Also collected will be the uterine volume, estimated by pelvic ultrasound or by pelvic MRI if available, the benign uterine pathology requiring the intervention (fibroid, adenomyosis, endometrial hyperplasia, bleeding), the general condition of the patient, the solidity of the vaginal suture, postoperative pain and use of analgesics, the possible occurrence of the above-mentioned complications and sexual disorders with the PISQ 12 scale. These data will be validated by the investigator who will sign the observation notebooks. Missing data will be justified.

### Statistical analysis

The included population will described globally and by group (vNOTES vs laparoscopic approach). The main analysis will be performed according to the intention-to-treat principle. The frequency of patients presenting with at least one complication will be estimated in each group, as well as the 95% confidence interval of this frequency. The effect of the surgical strategy will be estimated by the relative risk and the 95% confidence interval. The resulting p-value of Fisher’s exact test will also be given. As the secondary objectives are exploratory, no statistical test will be performed (i.e. no p-value will be estimated), according to the new NEJM recommendations.

For the qualitative secondary endpoints, the relative risk and its confidence interval will be given. For quantitative endpoints, differences in means will be calculated, as well as their 95% confidence intervals.

## Discussion

The available data from randomised controlled trials are insufficient to recommend the use of vNOTES for a hysterectomy in this case large volume uteri .

The first evaluations of vNOTES are encouraging and suggest a new era for pelvic surgery: less postoperative pain, fewer complications and facilitation of ambulatory care. Also the videoscopic assistance of the vNOTES is a pedagogical tool for the vaginal approach because the field of vision is no longer limited to the operator alone. Our study would be the first multicentric and academic study on vNOTES to focus specifically on large volume uteri , which are recognised as more difficult operations and prone to postoperative complications.

The expected benefits of this study are severalfold. For public health, the main objective is to reduce the length of hospitaliation. From the point of view of the patients, this study seeks a reduction in the length of hospitalisation, postoperative complications and postoperative pain. For the surgeon, it would improve visual comfort during surgery and the feeling of safety, as well as being an educational aid for teaching interns. The latter could effectively gain in visual comfort during surgery.

We hope that this large-scale, multicentric, randomised trial will lead to a better understanding of the approach to large uteri . Each year, several thousands of women undergo a hysterectomy in France; this study will support the evidence for optimal surgical care and better patient outcomes.
